# Supplementary material for: A Systematic Comparison of Antiandrogens Identifies Androgen Receptor Protein Stability as an Indicator for Treatment Response
Source: Life (Basel). 2021 Aug 25;11(9):874. doi: 10.3390/life11090874 (PMC8468615; doi:10.3390/life11090874)
Supplement: Supplementary file 1 [file life-11-00874-s001.zip › SFIG.pdf]

Figure S1: Cell line comparison of the influence of antiandrogens on the AR-mediated gene transactivation

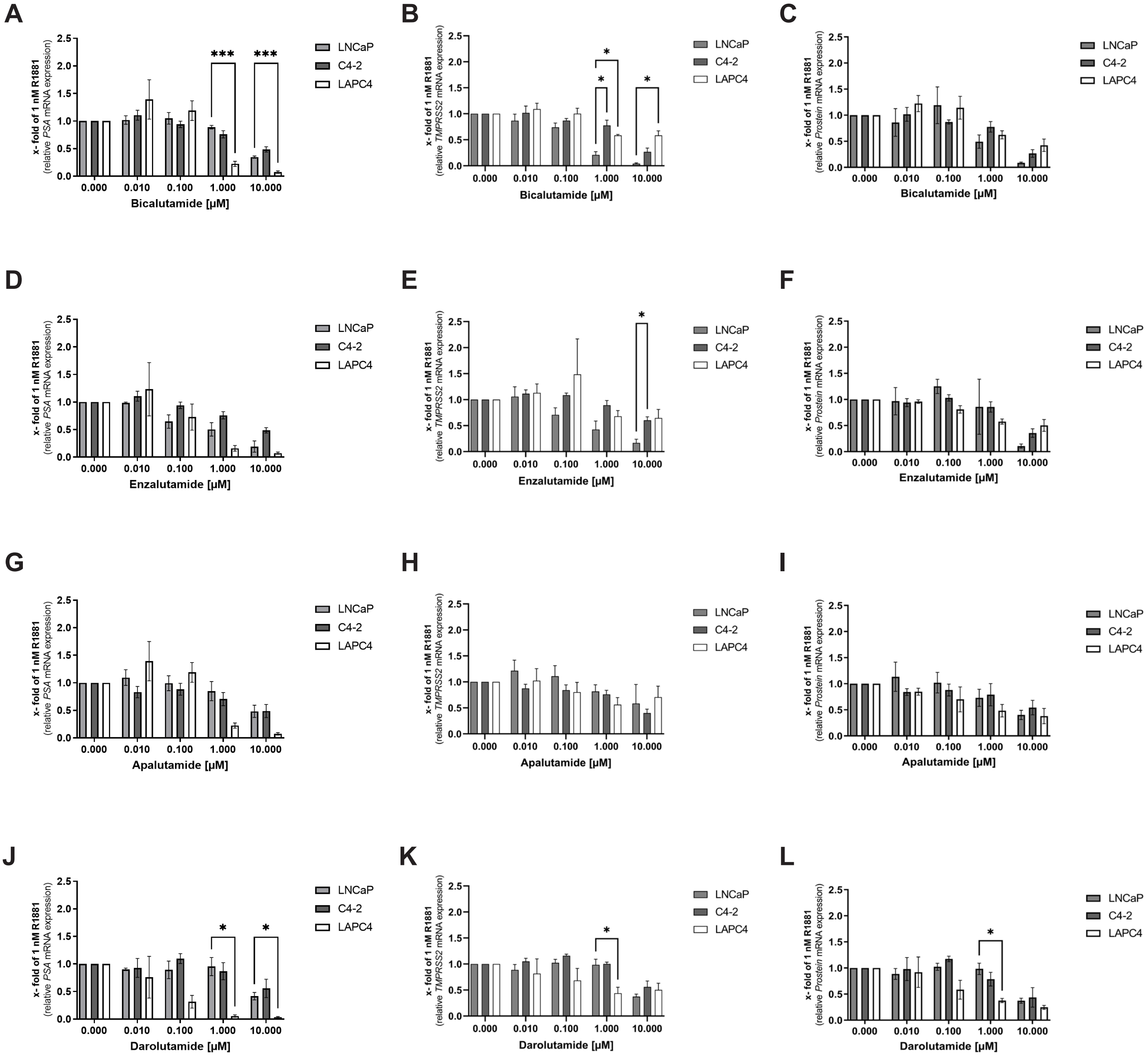

Figure S2: Nuclear und cytoplasmic fraction quality blots

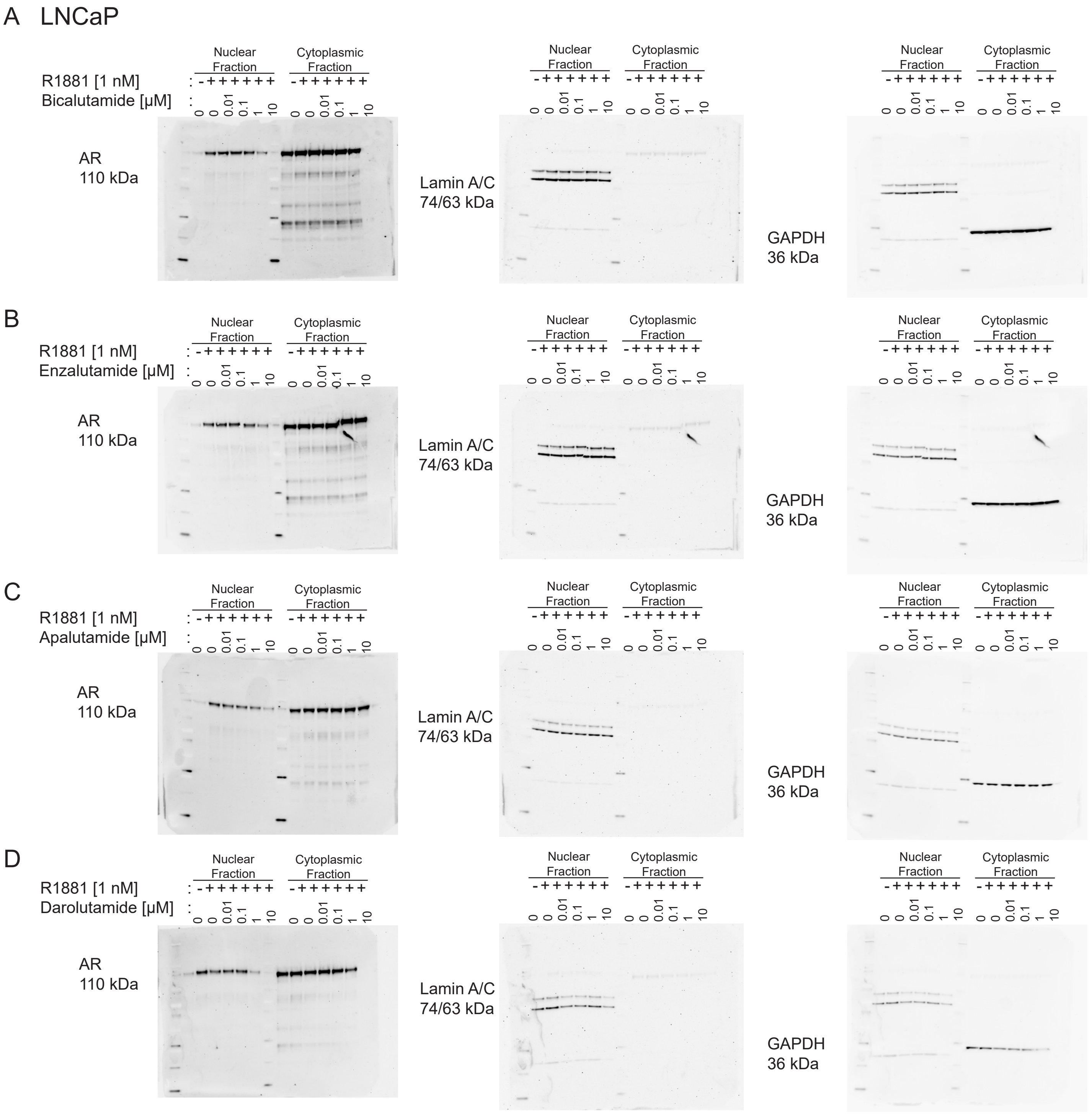

E

	Bicalutamide	Bicalutamide	Bicalutamide	Bicalutamide	Bicalutamide	Bicalutamide	Enzaltuamide	Enzaltuamide	Enzaltuamide	Enzaltuamide	Enzaltuamide	Enzaltuamide
	neg	pos	0.01	0.1	1	10	neg	pos	0.01	0.1	1	10
AR	946000	7030000	8400000	6790000	6230000	3290000	1050000	10600000	12300000	14000000	11400000	5100000
LAMIN AC	17900000	25600000	25500000	20600000	25400000	18400000	20500000	19400000	19800000	27500000	26100000	24700000
AR/LAMIN/A	0.052849162	0.274609375	0.329411765	0.32961165	0.245275591	0.178804348	0.051219512	0.546391753	0.621212121	0.509090909	0.436781609	0.206477733

	Apalutamide	Apalutamide	Apalutamide	Apalutamide	Apalutamide	Apalutamide	Darolutamide	Darolutamide	Darolutamide	Darolutamide	Darolutamide	Darolutamide
	neg	pos	0.01	0.1	1	10	neg	pos	0.01	0.1	1	10
AR	739000	7270000	5630000	5230000	3750000	1380000	1370000	11000000	5430000	6830000	8870000	2030000
LAMIN AC	8300000	9580000	9510000	8370000	9510000	8300000	8080000	10200000	4840000	5320000	7450000	3120000
AR/LAMIN/A	0.089036145	0.758872651	0.592008412	0.624850657	0.394321767	0.16626506	0.169554455	1.078431373	1.121900826	1.283834586	1.190604027	0.650641026

Figure S3: Nuclear und cytoplasmic fraction quality blots

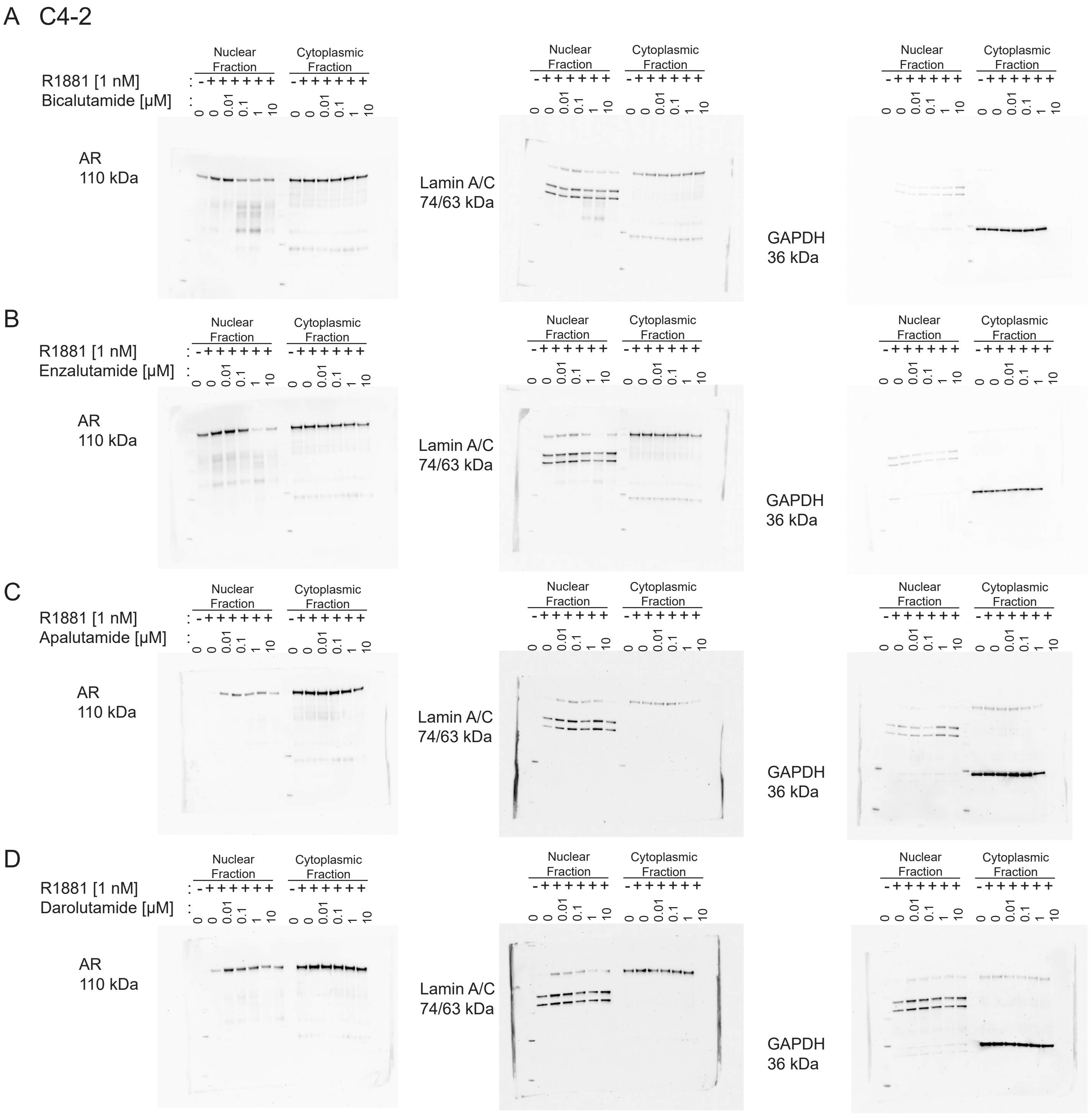

E

	Bicalutamide	Bicalutamide	Bicalutamide	Bicalutamide	Bicalutamide	Bicalutamide	Enzaltuamid	Enzaltuamid	Enzaltuamid	Enzaltuamid	Enzaltuamid	Enzaltuamide
	neg	pos	0.01	0.1	1	10	neg	pos	0.01	0.1	1	10
AR	13300000	34200000	38600000	32500000	32500000	14800000	8720000	39700000	38200000	27700000	29700000	14700000
LAMIN AC	35500000	32100000	34500000	26800000	29300000	31800000	36600000	48400000	40600000	37000000	53700000	46400000
AR/LAMIN/A	0.05284916	0.27460938	0.32941176	0.32961165	0.24527559	0.17880435	0.05121951	0.54639175	0.62121212	0.50909091	0.43678161	0.206477733

	Apalutamide	Apalutamide	Apalutamide	Apalutamide	Apalutamide	Apalutamide	Darolutamid	Darolutamid	Darolutamid	Darolutamid	Darolutamid	Darolutamide
	neg	pos	0.01	0.1	1	10	neg	pos	0.01	0.1	1	10
AR	13600000	60800000	19500000	40600000	22700000	8980000	13800000	57900000	68200000	26100000	28800000	13900000
LAMIN AC	97200000	134000000	61900000	77100000	58200000	57500000	76000000	89500000	91400000	59900000	61500000	46200000
AR/LAMIN/A	0.1399177	0.45373134	0.31502423	0.52658885	0.39003436	0.15617391	0.18157895	0.64692737	0.74617068	0.43572621	0.46829268	0.300865801

Figure S4: Nuclear und cytoplasmic fraction quality blots

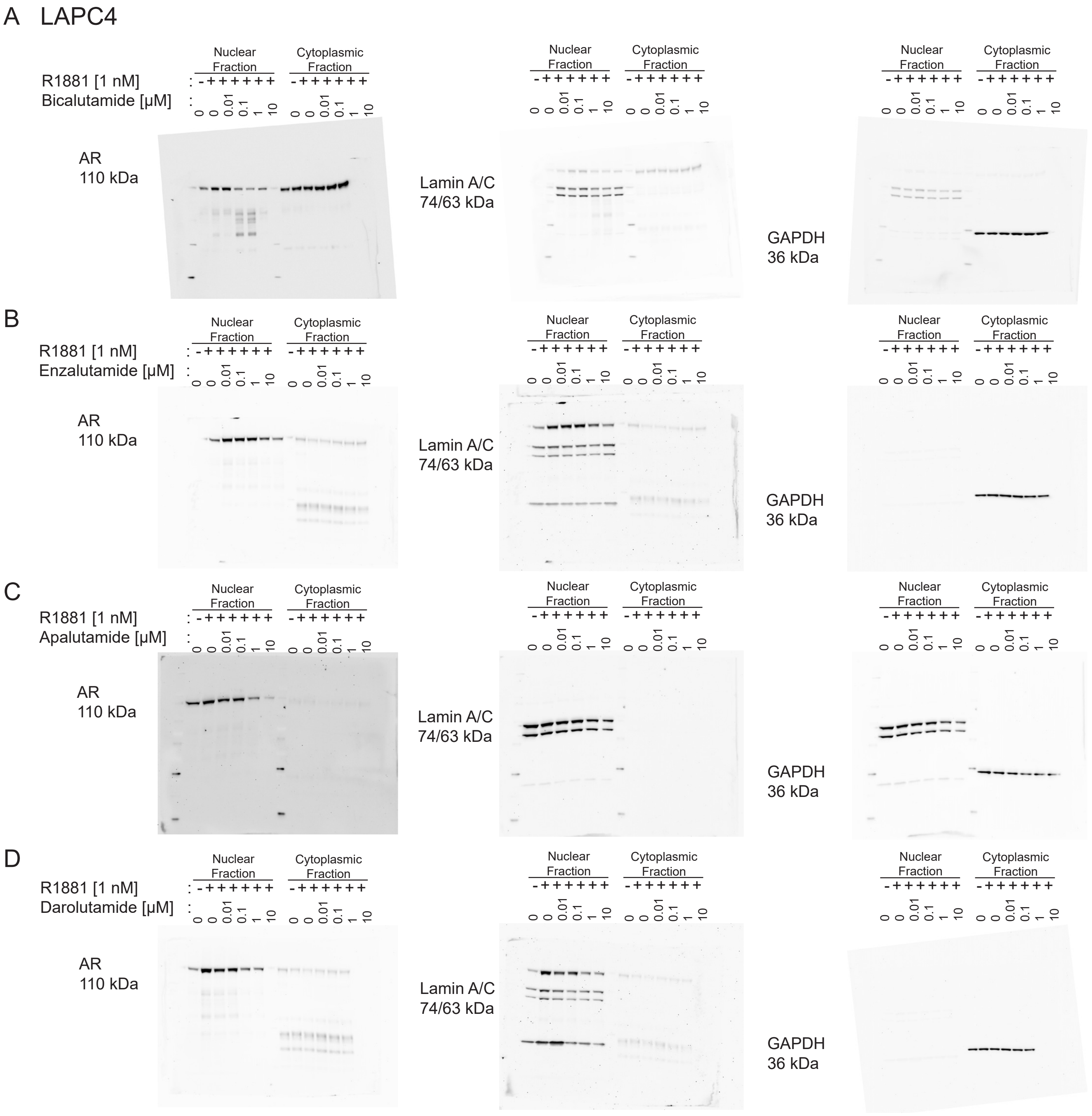

E

	Bicalutamide	Bicalutamide	Bicalutamide	Bicalutamide	Bicalutamide	Bicalutamide	Enzaltuamid	Enzaltuamid	Enzaltuamid	Enzaltuamid	Enzaltuamid	Enzaltuamide
	neg	pos	0.01	0.1	1	10	neg	pos	0.01	0.1	1	10
AR	3960000	11400000	6540000	20100000	6830000	8400000	1720000	688000	3830000	907000	2280000	103000
LAMIN AC	15400000	20800000	15500000	40000000	23500000	38300000	13500000	4540000	11100000	5040000	13000000	1150000
AR/LAMIN/A	0.25714286	0.54807692	0.42193548	0.5025	0.2906383	0.21932115	0.12740741	0.15154185	0.34504505	0.17996032	0.17538462	0.089565217

	Apalutamide	Apalutamide	Apalutamide	Apalutamide	Apalutamide	Apalutamide	Darolutamid	Darolutamid	Darolutamid	Darolutamid	Darolutamid	Darolutamide
	neg	pos	0.01	0.1	1	10	neg	pos	0.01	0.1	1	10
AR	2010000	3300000	2250000	2500000	1140000	442000	101000	1780000	2210000	1930000	2310000	961000
LAMIN AC	23700000	14500000	10000000	8910000	8040000	4460000	8810000	12500000	11300000	10700000	12800000	7390000
AR/LAMIN/A	0.08481013	0.22758621	0.225	0.28058361	0.14179104	0.09910314	0.01146425	0.1424	0.19557522	0.18037383	0.18046875	0.130040595

Figure S5: Densitometry analysis of AR protein levels in the cytoplasmic fraction and PSA protein levels after antiandrogen treatment

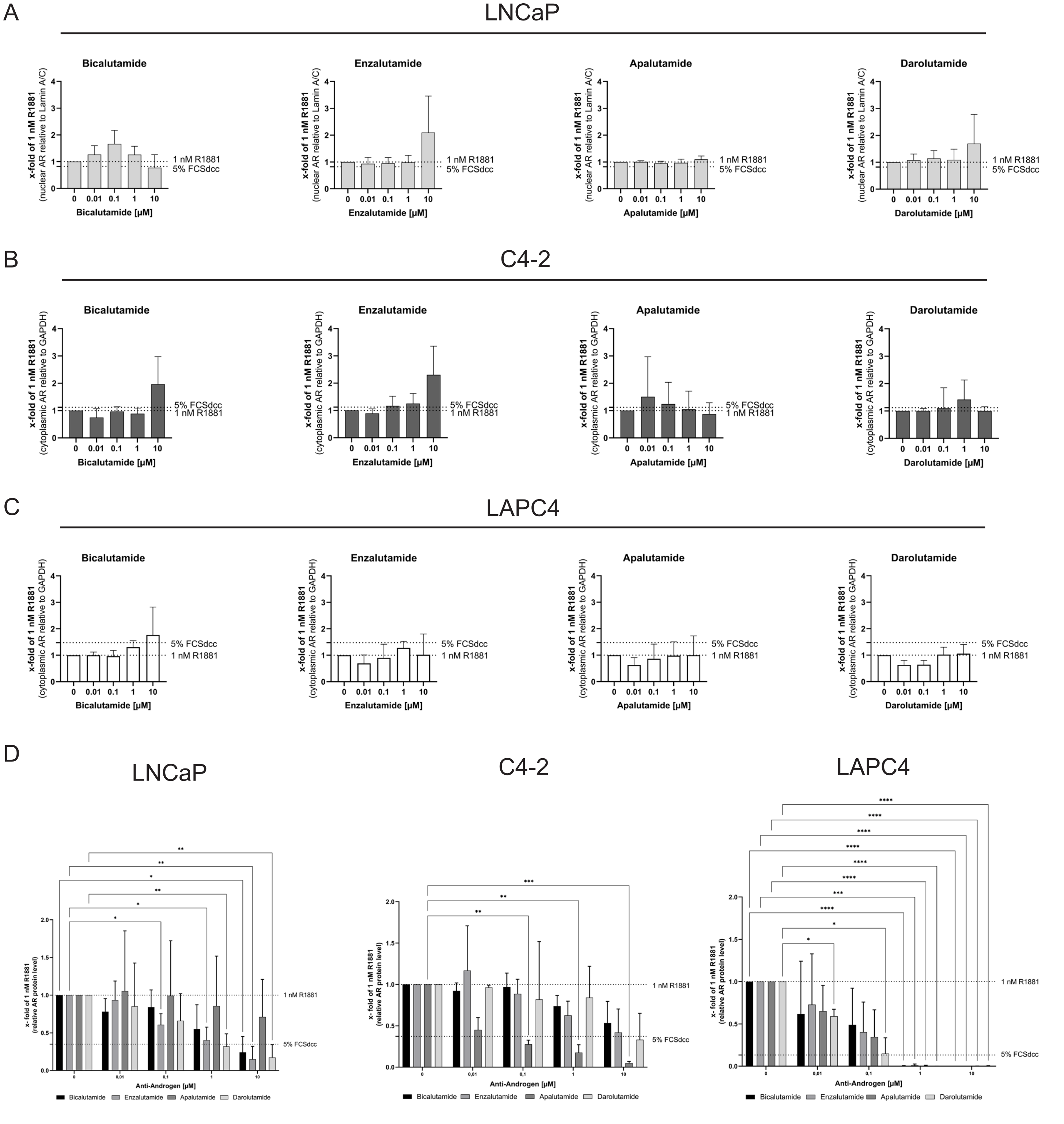

Figure S6: Uncropped western blot images

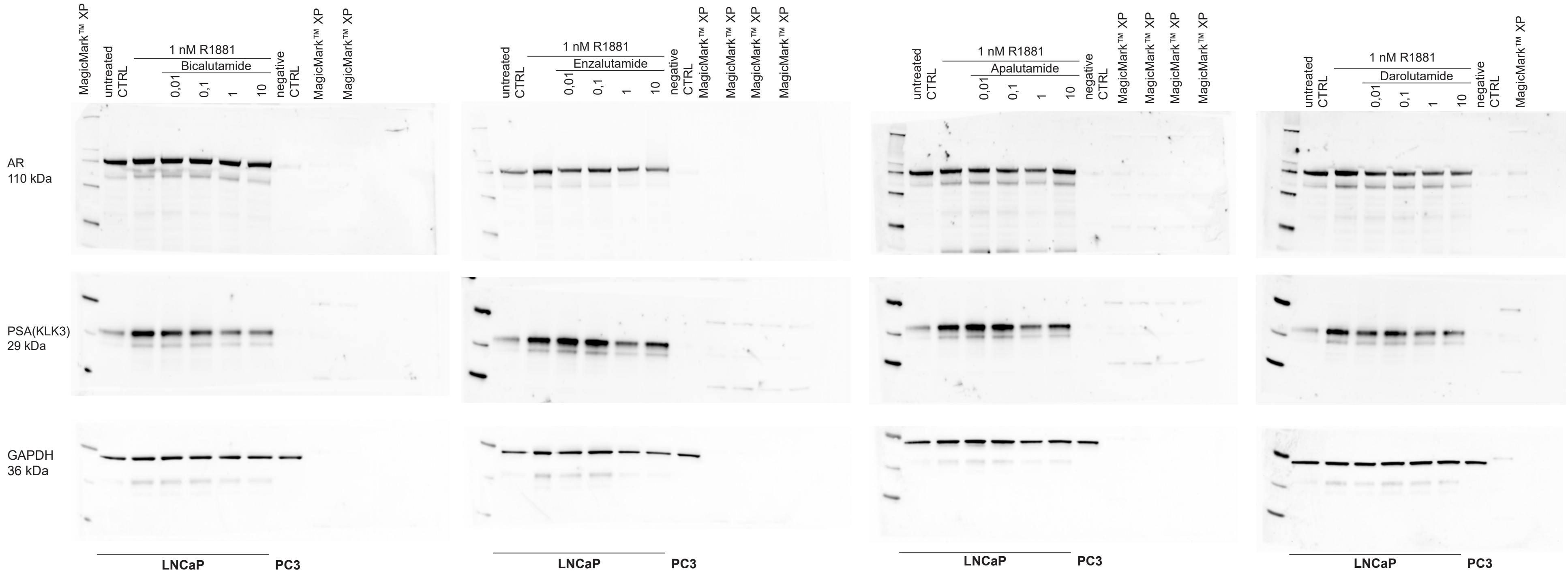

		1 nM R1881							1 nM R1881				
	untreated		Enzalutamide				negative	untreated		Bicalutamide			negative
	CTRL		0.01	0.1	1	10	CTRL	CTRL		0.01	0.1	1	10	CTRL
AR	73500000	1.85E+08	78800000	94700000	87400000	35200000	0	68800000	1.5E+08	1.67E+08	1.63E+08	1.2E+08	1.1E+08	0
PSA(KLK3)	22900000	67300000	50500000	42800000	24100000	28800000	0	14800000	67500000	42900000	71600000	18700000	1.6E+07	0
GAPDH	1.33E+08	1.47E+08	1.12E+08	1.41E+08	1.51E+08	1.44E+08	1.1E+07	1.2E+08	1.11E+08	1.24E+08	1.42E+08	1.41E+08	1.8E+08	1.4E+07
AR/GAPDH	0.55263	1.25850	0.70357	0.67163	0.57881	0.24444	0.00000	0.57333	1.35135	1.34677	1.14789	0.85106	0.62637	0.00000
PSA/GAPDH	0.17218	0.45782	0.45089	0.30355	0.15960	0.20000	0.00000	0.12333	0.60811	0.34597	0.50423	0.13262	0.08681	0.00000
	LNCaP						PC3	LNCaP						PC3

		1 nM R1881							1 nM R1881				
	untreated		Apalutamide				negative	untreated		Darolutamide			negative
	CTRL		0.01	0.1	1	10	CTRL	CTRL		0.01	0.1	1	10	CTRL
AR	40200000	92600000	1.04E+08	1.17E+08	65500000	1.31E+08	0	29200000	43000000	57900000	45200000	45700000	2.2E+07	0
PSA(KLK3)	15700000	67700000	87600000	81500000	30800000	47900000	0	7470000	42100000	25600000	34600000	19900000	1.7E+07	0
GAPDH	1.12E+08	87300000	1.5E+08	1.16E+08	51200000	1.01E+08	1.2E+07	1.08E+08	82400000	76400000	67800000	77200000	8.1E+07	9270000
AR/GAPDH	0.35893	1.06071	0.69333	1.00862	1.27930	1.29703	0.00000	0.27037	0.52184	0.75785	0.66667	0.59197	0.27518	0.00000
PSA/GAPDH	0.14018	0.77549	0.58400	0.70259	0.60156	0.47426	0.00000	0.06917	0.51092	0.33508	0.51032	0.25777	0.20885	0.00000
	LNCaP						PC3	LNCaP						PC3

Figure S7: Uncropped western blot images

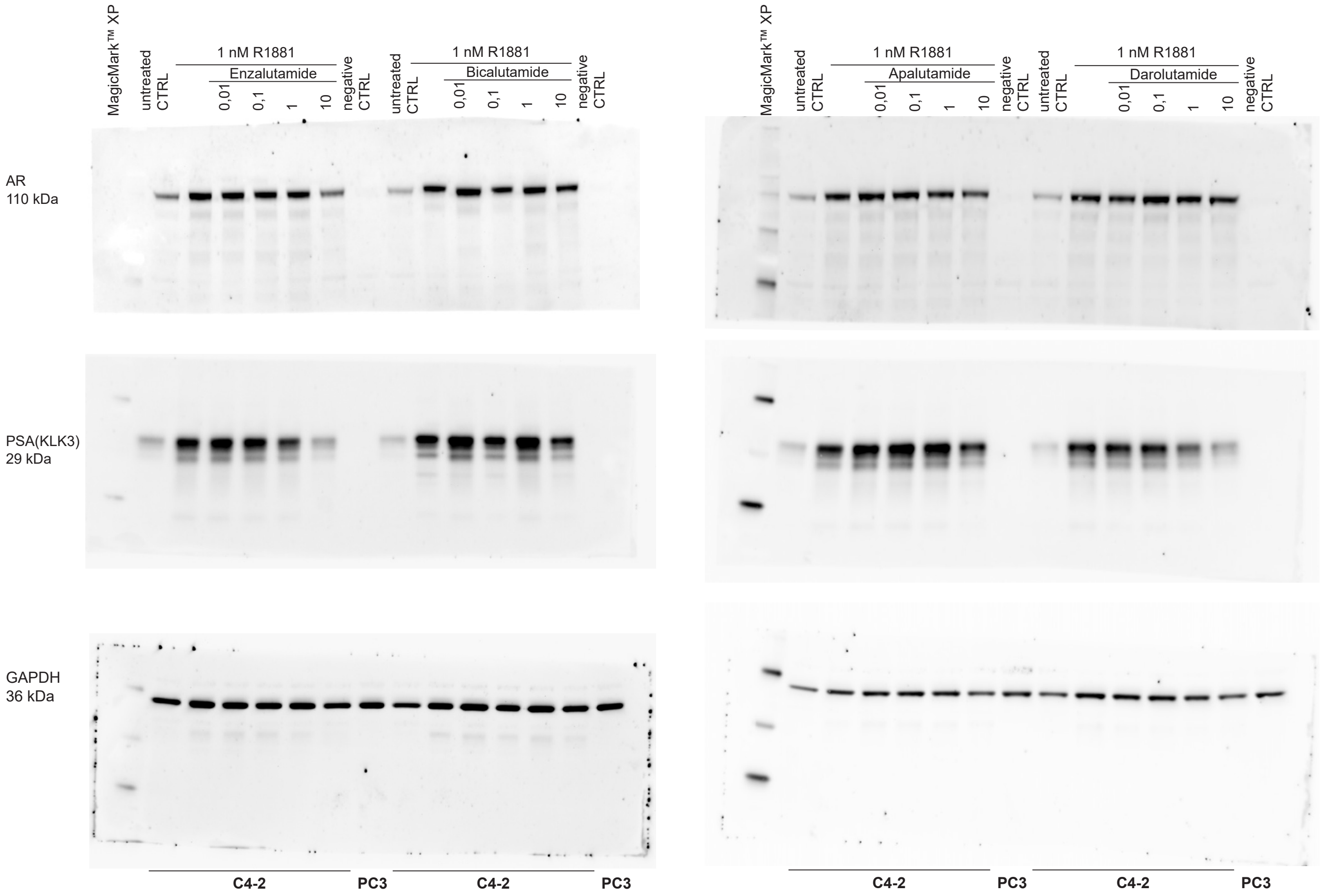

		1 nM R1881							1 nM R1881					
	untreated		Enzalutamide				negative	untreated		Bicalutamide				negati
	CTRL		0.01	0.1	1	10	CTRL	CTRL		0.01	0.1	1	10	CTRL
AR	10100000	32000000	25800000	24600000	23500000	12700000	0	6610000	22700000	30900000	19200000	25800000	2.1E+07	
PSA(KLK3)	7730000	29900000	32300000	30700000	21600000	8850000	0	4310000	42200000	46100000	28000000	42700000	2.3E+07	
GAPDH	8180000	13500000	12200000	10300000	11800000	7510000	1.1E+07	5870000	12500000	14700000	10800000	13100000	1E+07	1.4E+
AR/GAPDH	1.23472	2.37037	2.11475	2.38835	1.99153	1.69108	0.00000	1.12606	1.81600	2.10204	1.77778	1.96947	2.04950	0.000
PSA/GAPDH	0.94499	2.21481	2.64754	2.98058	1.83051	1.17843	0.00000	0.73424	3.37600	3.13605	2.59259	3.25954	2.27723	0.000
	C4-2						PC3	C4-2						PC3

		1 nM R1881							1 nM R1881					
	untreated		Apalutamide				negative	untreated		Darolutamide				negative
	CTRL		0.01	0.1	1	10	CTRL	CTRL		0.01	0.1	1	10	CTRL
AR	7330000	26900000	33300000	32300000	27400000	21400000	0	7890000	32100000	26900000	38800000	30800000	2.9E+07	0
PSA(KLK3)	8990000	50700000	67000000	74500000	56600000	34800000	0	5840000	56900000	52200000	39700000	22600000	1.9E+07	0
GAPDH	6140000	10300000	12000000	11800000	11400000	8030000	1.2E+07	8070000	15500000	12900000	13300000	9790000	7710000	9270000
AR/GAPDH	1.19381	2.61165	2.77500	2.73729	2.40351	2.66501	0.00000	0.97770	2.07097	2.08527	2.91729	3.14607	3.72244	0.00000
PSA/GAPDH	1.46417	4.92233	5.58333	6.31356	4.96491	4.33375	0.00000	0.72367	3.67097	4.04651	2.98496	2.30848	2.42542	0.00000
	C4-2						PC3	C4-2						PC3

Figure S8: Uncropped western blot images

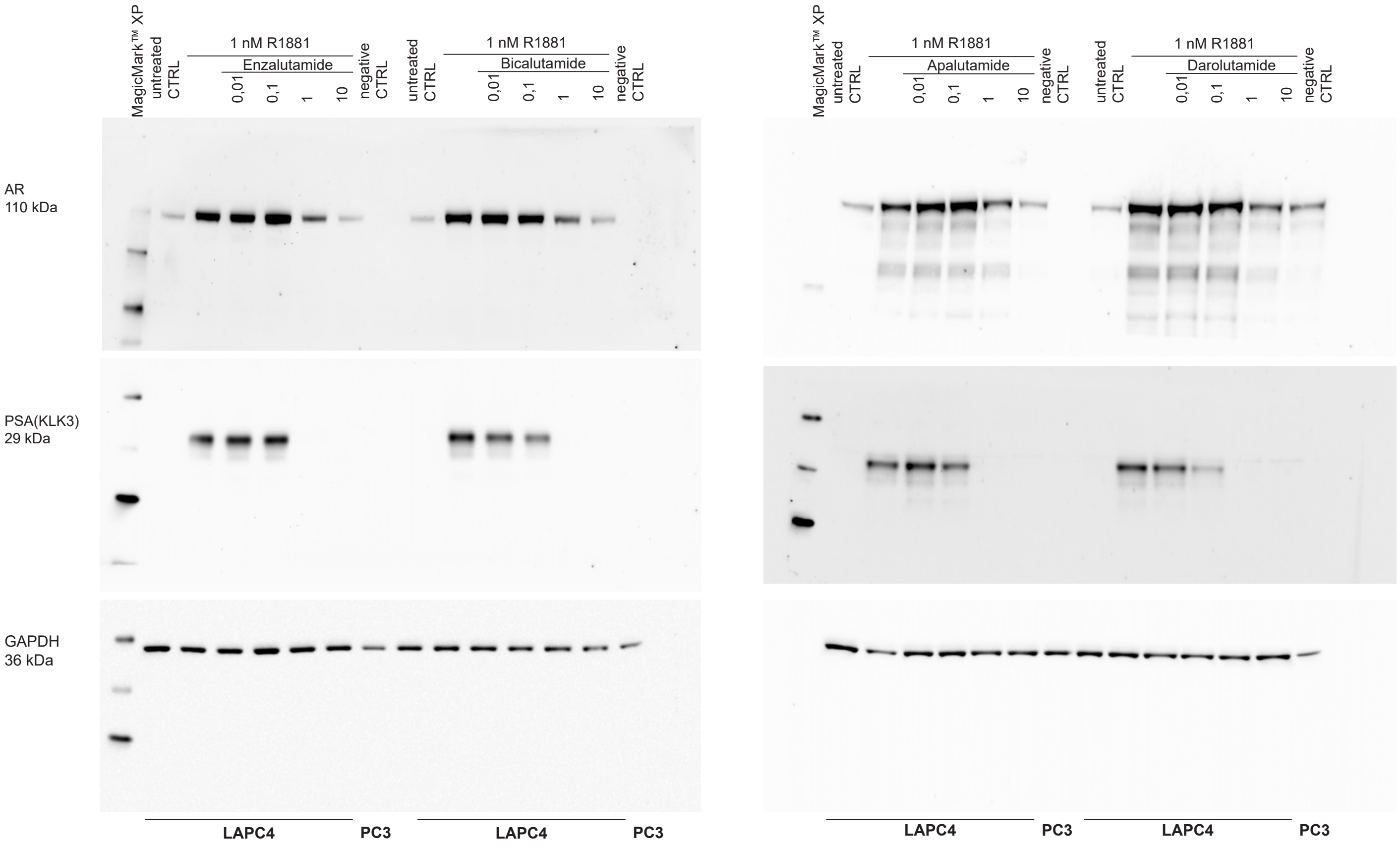

		1 nM R1881								1 nM R1881					
	untreated		Enzalutamide				negative	untreated		Bicalutamide				negati
	CTRL		0.01	0.1	1	10	CTRL	CTRL		0.01	0.1	1	10	CTRL
AR	2010000	39300000	43600000	63600000	9830000	1210000	13200	1870000	38000000	50000000	36700000	12100000	2360000	5000
PSA(KLK3)	1780	59500000	78100000	77600000	91400	2440	187	0	82200000	62100000	40700000	69300	0	
GAPDH	20300000	14200000	14300000	21900000	14400000	12500000	5330000	10100000	11300000	10300000	8780000	9150000	6690000	33800
AR/GAPDH	0.09901	2.76761	3.04895	2.90411	0.68264	0.09680	0.00248	0.18515	3.36283	4.85437	4.17995	1.32240	0.35277	0.147
PSA/GAPDH	0.00009	4.19014	5.46154	3.54338	0.00635	0.00020	0.00004	0.00000	7.27434	6.02913	4.63554	0.00757	0.00000	0.000
	LAPC4						PC3	LAPC4						PC3

		1 nM R1881								1 nM R1881					
	untreated		Apalutamide				negative	untreated		Darolutamide				negative
	CTRL		0.01	0.1	1	10	CTRL	CTRL		0.01	0.1	1	10	CTRL
AR	560000	4170000	7090000	7120000	2730000	381000	0	308000	8920000	8270000	6070000	1890000	1450000	0
PSA(KLK3)	265	5590000	7830000	4390000	0	0	0	0	6980000	5760000	1280000	33000	0	0
GAPDH	55000000	21300000	41200000	40900000	30900000	33100000	2.9E+07	36700000	44000000	34500000	33700000	33500000	4.5E+07	1E+07
AR/GAPDH	0.01018	0.19577	0.17209	0.17408	0.08835	0.01151	0.00000	0.00839	0.20273	0.23971	0.18012	0.05642	0.03208	0.00000
PSA/GAPDH	0.00000	0.26244	0.19005	0.10733	0.00000	0.00000	0.00000	0.00000	0.15864	0.16696	0.03798	0.00099	0.00000	0.00000
	LAPC4						PC3	LAPC4						PC3

Figure S9: Uncropped western blot images

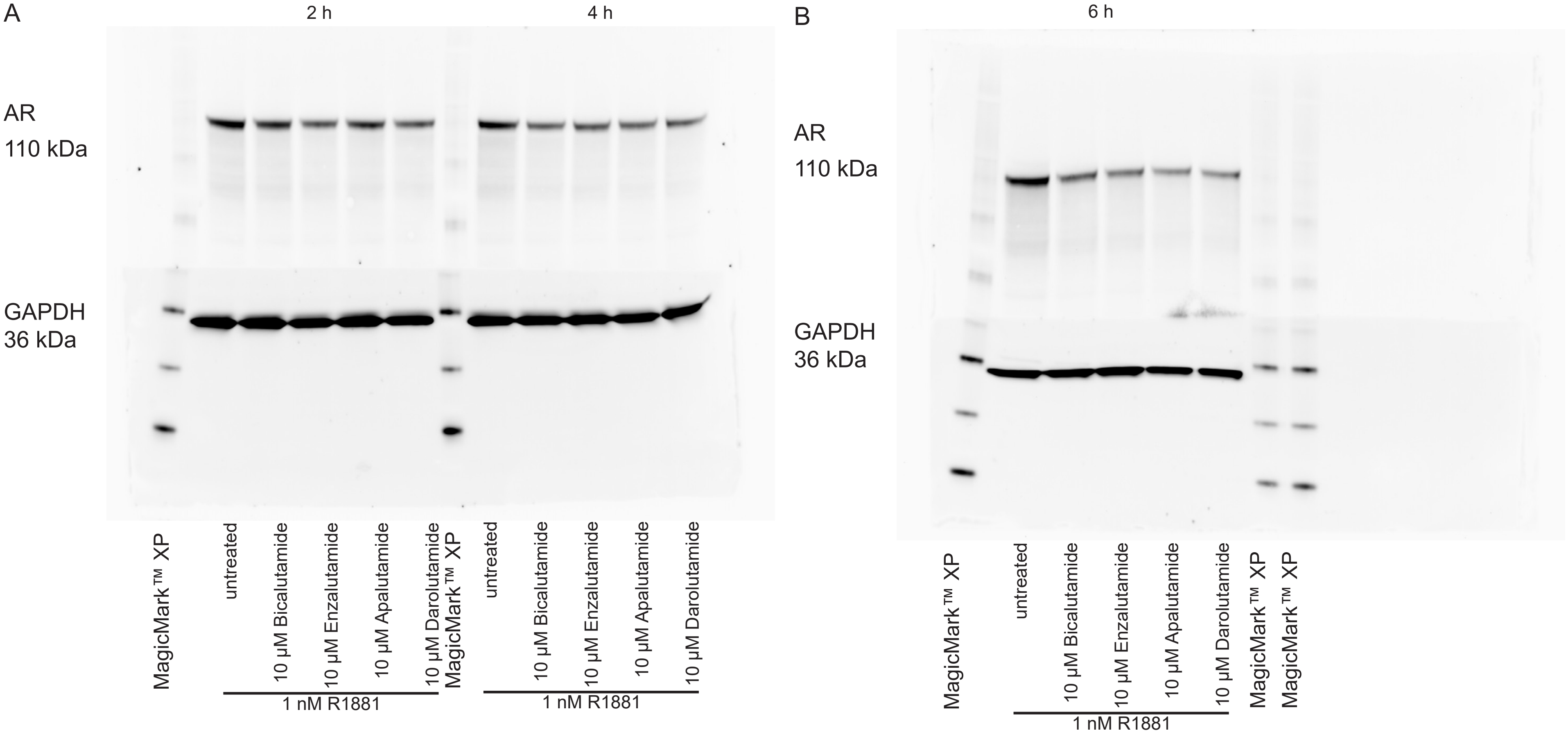

	2 h				
	untreated	10 µM Bicalutamide	10 µM Enzalutamide	10 µM Apalutamide	10 µM Darolutamide
AR	39300000	31400000	21600000	28800000	28000000
GAPDH	168000000	166000000	142000000	176000000	159000000
Ratio	0.233928571	0.189156627	0.152112676	0.163636364	0.176100629
	4 h				
	untreated	10 µM Bicalutamide	10 µM Enzalutamide	10 µM Apalutamide	10 µM Darolutamide
AR	35000000	20700000	22700000	21300000	24800000
GAPDH	162000000	148000000	149000000	132000000	150000000
Ratio	0.216049383	0.139864865	0.152348993	0.161363636	0.165333333
	6 h				
	untreated	10 µM Bicalutamide	10 µM Enzalutamide	10 µM Apalutamide	10 µM Darolutamide
AR	45800000	24100000	20200000	14400000	15600000
GAPDH	164000000	144000000	152000000	99500000	124000000
Ratio	0.279268293	0.167361111	0.132894737	0.144723618	0.125806452

Figure S10: Uncropped western blot images

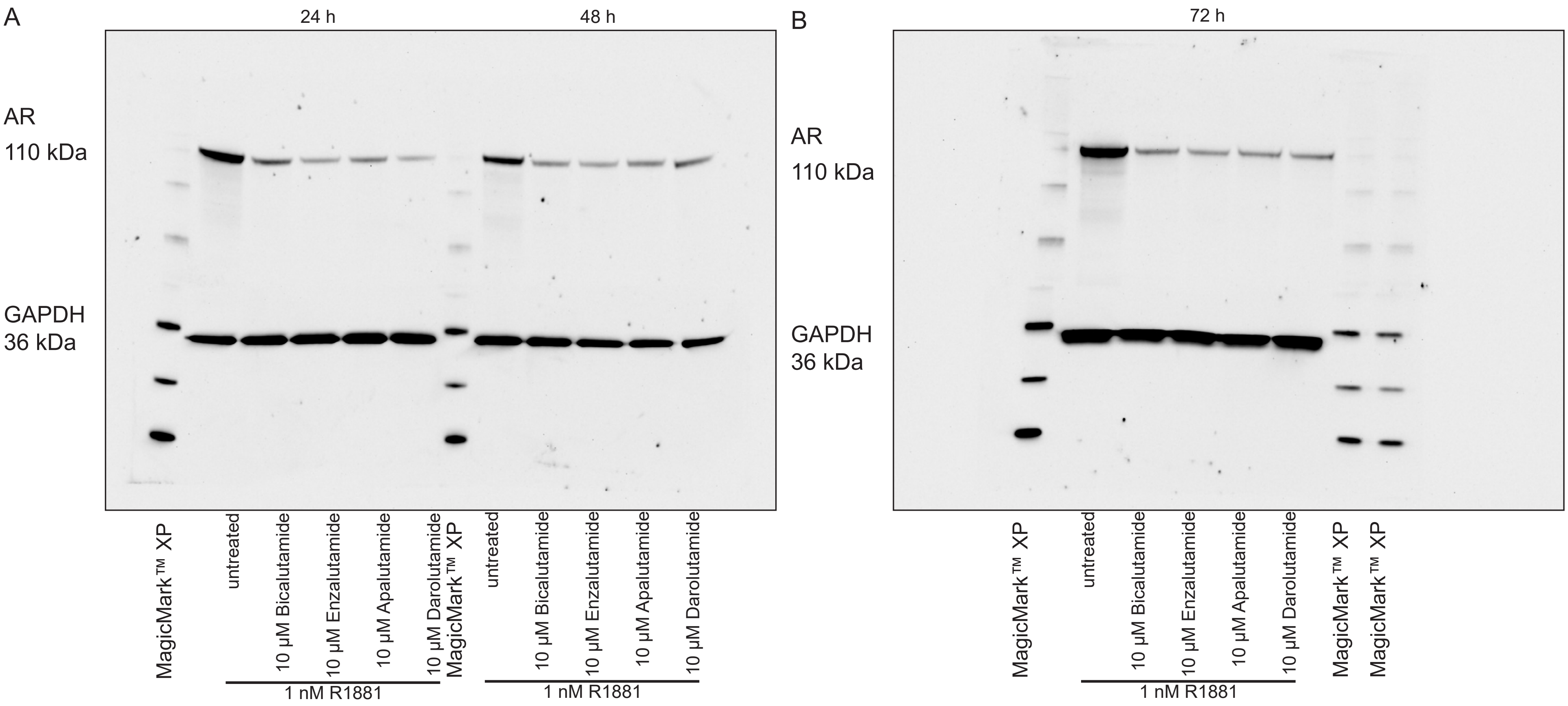

	2 h				
	untreated	10 µM Bicalutamide	10 µM Enzalutamide	10 µM Apalutamide	10 µM Darolutamide
AR	9130000	1700000	659000	898000	403000
GAPDH	28600000	39100000	43700000	55500000	36600000
Ratio	0.319230769	0.043478261	0.015080092	0.01618018	0.011010929
	4 h				
	untreated	10 µM Bicalutamide	10 µM Enzalutamide	10 µM Apalutamide	10 µM Darolutamide
AR	3950000	1040000	760000	1130000	1680000
GAPDH	25800000	22900000	15900000	20000000	15300000
Ratio	0.153100775	0.045414847	0.047798742	0.0565	0.109803922
	6 h				
	untreated	10 µM Bicalutamide	10 µM Enzalutamide	10 µM Apalutamide	10 µM Darolutamide
AR	14900000	1220000	825000	1060000	1270000
GAPDH	96700000	57900000	53800000	53600000	67300000
Ratio	0.154084798	0.021070812	0.015334572	0.019776119	0.018870728
